# A dataset for detecting walking, grazing, and resting behaviors in free-grazing cattle using IoT collar IMU signals

**DOI:** 10.3389/fvets.2025.1630083

**Published:** 2025-08-29

**Authors:** David Morales-Vargas, Miguel Guarda-Vera, Daniel Iglesias-Quilodrán, David Cancino-Baier, Carlos Muñoz-Poblete

**Affiliations:** ^1^Departamento de Ingeniería Eléctrica, Universidad de La Frontera, Temuco, Chile; ^2^Magíster en Ciencias de la Ingeniería, Universidad de La Frontera, Temuco, Chile; ^3^Facultad de Ciencias Agropecuarias y Medioambiente, Universidad de La Frontera, Temuco, Chile

**Keywords:** standardized dataset, behaviors, cattle, lameness, animal welfare, Internet of Things (IoT), IMU

## Abstract

In this study, we developed a dataset of behaviors associated with lameness in dairy cows. The data collection utilized IoT collars that were placed around the necks of 10 dairy cows. This publicly available dataset contains 441 labeled behaviors, amounting to over 7 h of recording time. It includes acceleration data referenced in both body and world frames, as well as gyroscope signals, which facilitated the extraction of 112 relevant features for classifying key behaviors such as walking, grazing, and resting through machine learning algorithms. To enhance model performance and reduce feature dimensionality, automatic feature selection techniques were applied before classification. The dataset's effectiveness was assessed using various classification models, including Support Vector Machines (SVM), Logistic Regression, Decision Trees, and Random Forests. Results indicated that signals referenced to the body frame yielded better behavior discrimination, achieving a maximum macro F1-score of 0.9625 with the SVM model. This public dataset can facilitate early lameness detection by enabling accurate classification of behavior patterns.

## 1 Introduction

In contemporary livestock production, the optimization of animal welfare and the efficient management of herds are essential for enhancing the profitability and sustainability of dairy farming operations. Among the most critical challenges that farmers encounter are the timely detection of estrus and the early identification of health issues, such as lameness, both of which significantly affect livestock productivity. Ineffective management of these conditions can result in substantial economic losses, characterized by low reproduction rates, diminished milk yield, and increased veterinary expenses (1–4).

Numerous studies have established the correlation between lameness and cow behavior, even prior to the visual manifestation of lameness (5). Lame cows typically display reduced feeding and walking activity, alongside an increase in lying time when compared to their healthy counterparts (6–9).

Walking, grazing, and resting constitute fundamental behaviors that serve as indicators of dairy cows' overall health and welfare. Variations in these activities represent some of the earliest signs of lameness, often detectable before the appearance of visible clinical symptoms. The act of walking is particularly relevant, as lame cows generally exhibit diminished locomotor activity due to pain or discomfort (8). Similarly, grazing behavior, which is intricately associated with feeding time and frequency, declines significantly in lame cows, reflecting both their decreased appetite and the physical strain associated with foraging (6, 7). In contrast, resting time tends to increase as a compensatory behavior, allowing cows to alleviate pressure on affected limbs (9). Consequently, the continuous and automated monitoring of these three behaviors offers critical insights into early lameness detection and supports timely intervention strategies aimed at safeguarding animal welfare and enhancing farm productivity.

The advancement of emerging technologies, especially the Internet of Things (IoT), has facilitated the deployment of automated solutions for continuously and precisely monitoring animal behavior. Recent reviews have underscored the efficacy of wearable sensors in classifying livestock behavior, such as accelerometers and Inertial Measurement Units (IMUs). When integrated with machine learning algorithms, these technologies provide robust tools for detecting lameness (10, 11). Daily activity patterns and specific movement behaviors exhibited by cows are closely correlated with their health and reproductive status (12). Additionally, other IoT-based methodologies, such as vision systems, have exhibited substantial potential. For instance, research conducted by Van Hertem et al. (13) utilized automatically registered three-dimensional video data for the early detection of lameness in dairy cows, thereby demonstrating the significance of continuous behavioral analysis.

In dairy farms using grazing systems, where cows feed directly from pastures, detecting lameness and other diseases is challenging due to the vast territory and the large number of livestock. Grazing systems are commonly used in Southern Hemisphere countries such as New Zealand, Australia, Argentina, Uruguay, Chile, and Brazil.

The literature reveals several public datasets dedicated to the detection of cattle movements. One notable example is CowScreeningDB, proposed by Ismail et al. (10), which is a multisensor dataset created from data collected from 43 dairy cows. In addition to making the dataset publicly accessible, the authors present a machine learning technique based on Support Vector Machines (SVM) designed to classify cows as healthy or lame. This methodology achieves an average accuracy rate of 77% in the best cases. However, this dataset does not include specific labels for normal walking or lameness events. Instead, it provides continuous data collection for 7 h, corresponding to the daily activity of healthy and lame cows. Furthermore, data were collected on cows confined to at least 10 square meters.

Haladjian et al. (11) proposed a sensor-based system for detecting lameness in dairy cattle. The dataset used to train the classification algorithm was made available to facilitate developing and validating alternative methods to improve cow welfare. However, the dataset construction did not include naturally lame cows; instead, lameness was simulated by placing a plastic block on the outer claw of the left or right hind hoof. Employing an SVM algorithm, an average accuracy of 91.1% was achieved. Ito et al. (14) also posted a dataset that includes triaxial accelerometer measurements with thirteen labeled behaviors. These data were collected using a Kionix KX122-1037 ±2g, 16-bit accelerometer placed on the neck of six Japanese black cows at a farm belonging to Shinshu University in Nagano, Japan. Data were collected over two days, allowing the cows to roam freely in a pasture field and farm pens, while being recorded with Sony FDR-X3000 4K video cameras. A total of 197 minutes of data were labeled, covering thirteen distinct behaviors. This dataset was utilized by Russel et al. (15), who, through a deep learning model, achieved the following performance metrics: in the worst case, an accuracy of 0.98, precision of 0.73, recall of 0.88, and an F1-score of 0.76 were obtained. In the best case, they reached values of 1.00 accuracy, 1.00 precision, 0.98 recall, and 0.99 F1-score.

A common aspect of the studies by Ismail et al. (10) and Haladjian et al. (11) is that data collection occurs in confined or controlled environments, and both employ methodologies involving measurement devices attached to the cows' legs. Since these studies took place in confined spaces, the results might differ from those obtained in free-grazing systems, where monitoring behavior is particularly challenging due to environmental variability and limited animal visibility (16). On the other hand, while Ito et al.'s (14) dataset is obtained in open spaces and farm pens, it only includes linear acceleration measurements in the body reference frame. Furthermore, behavior labeling was based on video recordings that were manually captured, a process which may influence cow behavior due to the presence of human operators during recording. It lacks gyroscope measurements, whereas in Liang et al. (17), the features extracted from the gyroscope have the highest permutation importance in movement classification.

This highlights the need for a more comprehensive dataset in free-grazing systems, incorporating new variables for behavior identification. Additionally, a more reliable system for recording events during dataset creation would improve the accuracy and robustness of the dataset.

A notably relevant study was conducted in Norway by Versluijs et al. (18), who monitored free-ranging beef cattle in rugged, forested pasture areas using GPS collars equipped with tri-axial accelerometers. Their work is notable not only for the natural grazing context but also for the preprocessing techniques explored, including smoothing window selection and orientation correction using a world reference frame. Despite testing models with and without orientation correction, their findings showed similar performance between both approaches, suggesting that the effect of correction may depend on the collar fit or terrain. This study serves as a reference for the real-world application of accelerometry in open grazing environments, supporting the relevance of our approach.

Building upon these insights, our study seeks to overcome limitations reported in earlier works conducted in confined or semi-controlled environments, unlike the natural settings addressed by Versluijs et al., where sensor placement and environmental complexity are often limited. By incorporating acceleration measurements in both body and world reference frames and collecting data in open grazing conditions, we aim to enhance behavior classification and provide a more realistic and generalizable dataset. These latter measurements could improve the detection of behaviors such as walking (19). Additionally, angular velocity measurements are included to identify behaviors such as walking, grazing, and resting. Reliability and reproducibility of the results are ensured through rigorous documentation of the data collection and labeling process. Unlike the methodologies presented in (10) and (11), data collection is conducted on grazing dairy cows using an IoT collar and a high-range Pan-Tilt-Zoom (PTZ) camera with night vision. The camera will be mounted on a 9-meter-high pole with a GPS tracking system to obtain corresponding videos when cows are in an 80-hectare grazing environment.

To address the challenges of behavior monitoring in free-grazing dairy systems, this work introduces a novel and fully documented dataset of annotated cow behaviors collected in open pasture conditions using wearable sensors and video validation. The dataset aims to support the development and testing of machine learning models for behavior recognition and early detection of lameness-related patterns in real-world environments.

The key contributions of this work are summarized as follows:

1. A publicly available dataset for detecting behaviors associated with lameness, including accelerometer and gyroscope data collected from grazing dairy cows in southern Chile, along with an example script demonstrating how to implement a machine learning algorithm using these data;
2. Inclusion of acceleration and gyroscope data in body and world reference frames;
3. Implementation and testing of various machine learning algorithms using the behavior dataset, demonstrating its utility in classification tasks.

## 2 Materials and methods

**Figure 1:**
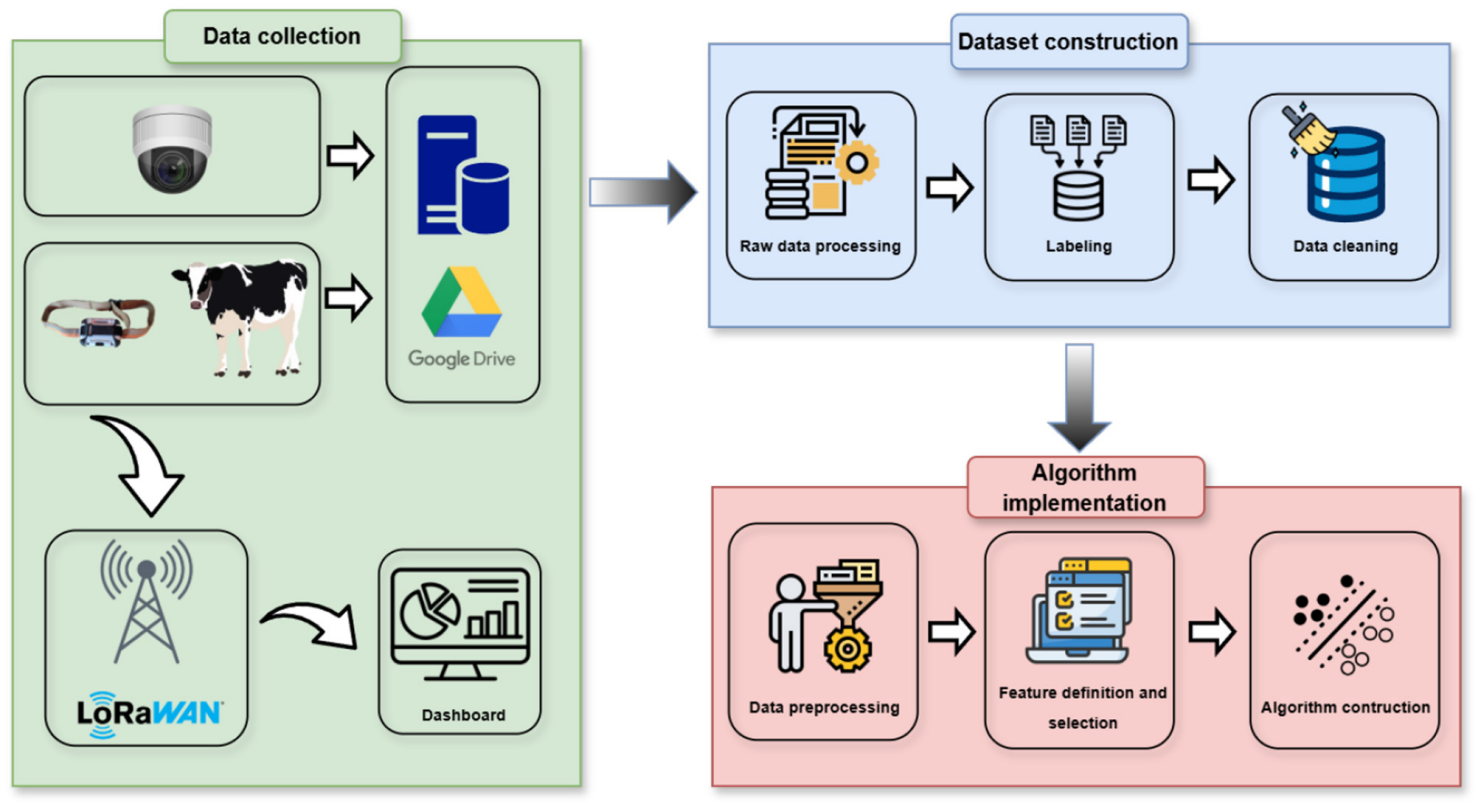
Diagram of the proposed methodology.

This section describes the proposed methodology, which is illustrated in Figure 1. As shown, it is divided into three main stages, detailed below.

### 2.1 Data collection

#### 2.1.1 Field details

**Figure 2:**
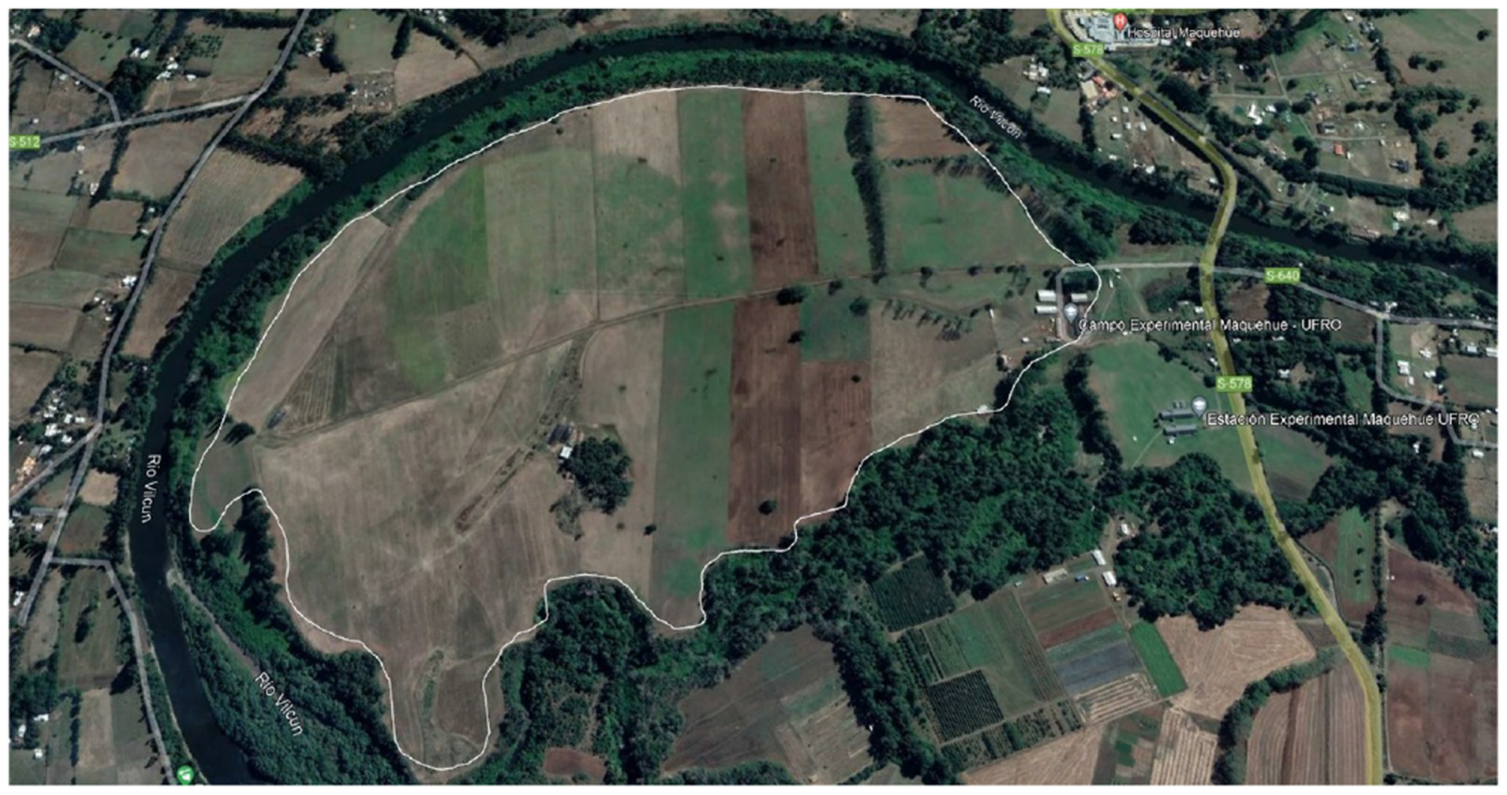
Location of the Maquehue experimental field. The white line indicates the boundary of the LoRaWAN network (maps data: Google Earth, © 2025 / Maxar Technologies).

The data acquisition process was carried out at a dairy farm located in the Maquehue Experimental Farm (see Figure 2), owned by the Universidad de La Frontera in Temuco, Chile. It is situated 17 km south of Temuco, in the Freire municipality (Lat: −38.8379, Long: −72.6938). This farm has a total of 120 Holstein Friesian cows that are raised on open pasture with access to food and water.

#### 2.1.2 Data and video collection system

**Figure 3:**
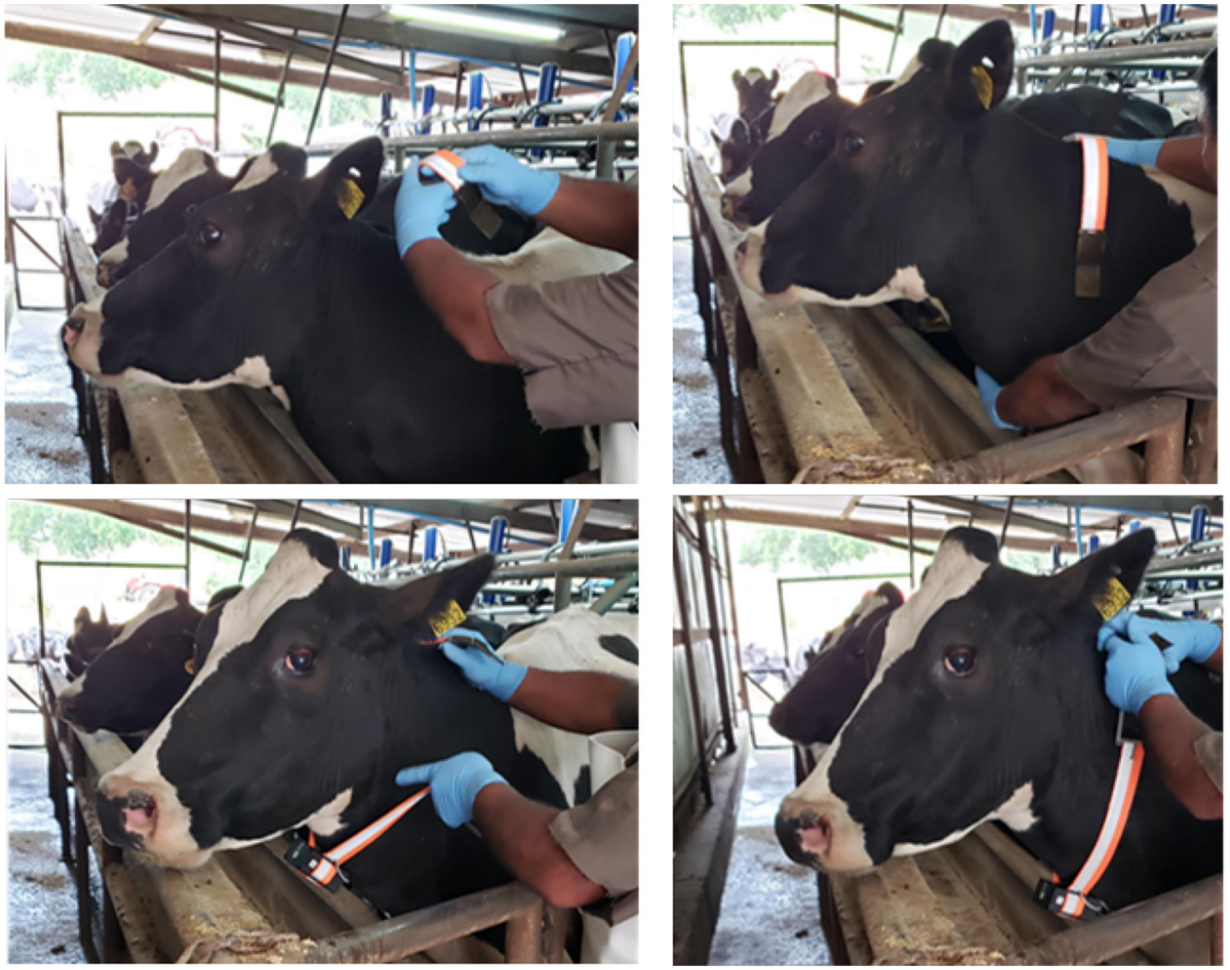
Photographs showing the installation of the IoT collar.

Data were collected using IoT collars installed around the animals' necks, as illustrated in Figure 3. Each collar integrates two IMUs: the MPU9250 and the BNO055, both sampling at 10 Hz. BNO055 was included in the collar, as it provides orientation in quaternion format, allowing the calculation of acceleration in the world reference frame, while MPU9250 is a low-cost sensor alternative. The collars also include GPS functionality, and both location data and battery status are transmitted via a LoRa network for real-time visualization on a Grafana dashboard. Additional components of each device include a Wireless Stick V3 microcontroller, an SD card slot for local data storage, and a Real-Time Clock (RTC).

**Figure 4:**
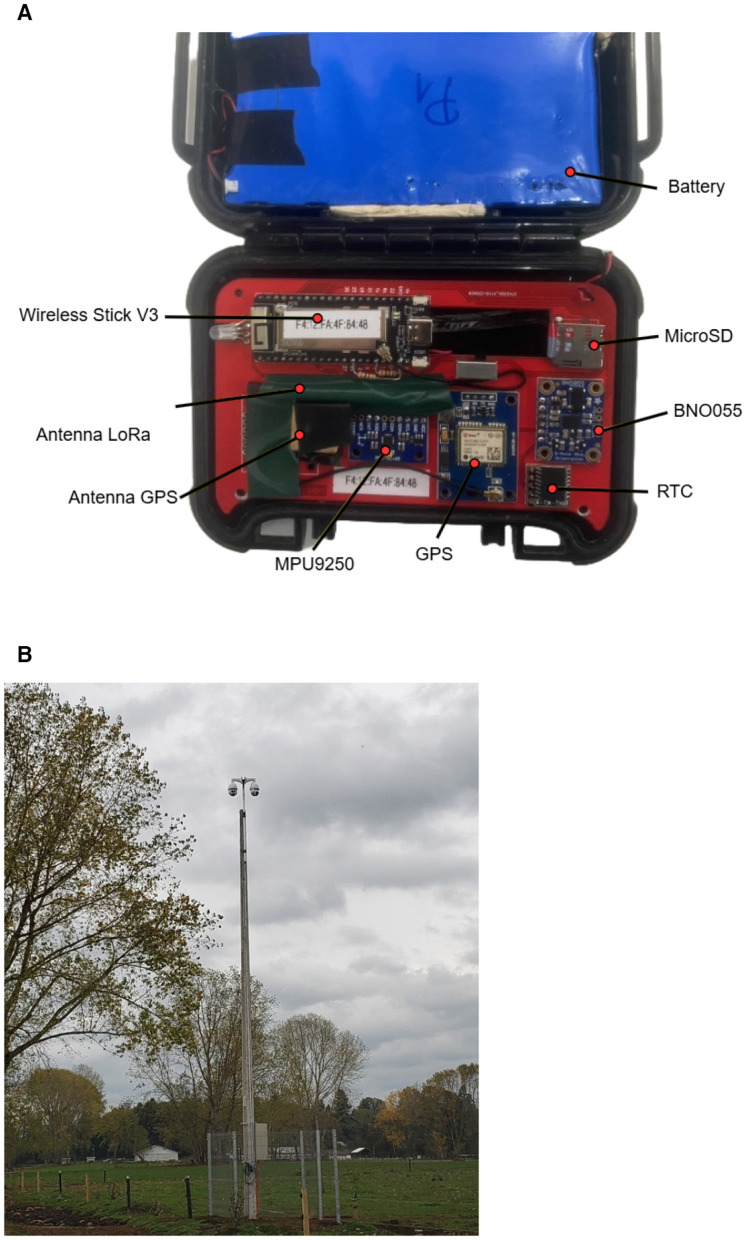
Data collection system setup. **(A)** IoT collar components used for data collection. **(B)** PTZ cameras used for monitoring.

Data recorded by the collar are stored in Comma-Separated Values (CSV) files on a microSD card, which is removed at the end of the measurement period for data storage. Figure 4A provides a detailed view of the data collection device components.

To validate cow behaviors, a system of PTZ cameras (model IP DAHUA 2MP SD6AL230F-HNI) records the pasture throughout the day. This system includes automatic monitoring of general cattle behavior (20). Recent modifications enable the system to individually locate cows equipped with IoT collars via their GPS coordinates (21). Video recordings obtained from these cameras are stored on a Network Video Recorder (NVR) for a limited time before being transferred to a server. Figure 4B shows a photo of the PTZ cameras used for video acquisition.

### 2.2 Dataset construction

#### 2.2.1 Data processing

At this stage, the relevant columns for the dataset are processed. These correspond to the variables recorded by the BNO055 IMU, which include linear acceleration, angular velocity, and quaternion-based orientation. Additionally, a column is added for acceleration in the world frame (WF). This transformation expresses acceleration relative to the Earth's surface, making both acceleration and orientation measurements independent of the sensor's posture or position on the animal. The procedure to convert accelerations from the body frame (BF) to the world frame using a rotation matrix can be found in Muñoz-Poblete et al. (19). Furthermore, the variables measured by the MPU9250 IMU, including linear acceleration, gyroscope, and magnetometer readings, are also included.

To perform behavior labeling, it is necessary to synchronize the signal timestamps with the video timestamps. For this purpose, a time column (date and time) is generated to facilitate the labeling process. Since the measurements provided by the IMUs are raw values, these need to be converted to their corresponding physical units. This is achieved using a linear scaling method to transform the data into a defined range. In this context, this involves multiplying each raw value in the CSV files by a scaling factor, which consists of the resolution value and the sensor's sensitivity. The equations for scaling raw data for the accelerometer and gyroscope are as follows:


(1) ab=ar·Ra2ba ·9.807

(2) wb=wr·Rw2bw

where ^*b*^*a* represents the acceleration data in *m*/*s*^2^, and ^*b*^*w* corresponds to the gyroscope measurements in °/*s*. *a*_*r*_ is the raw accelerometer value, and *w*_*r*_ is the raw gyroscope value. *R*_*a*_ and *R*_*w*_ denote the measurement ranges of the accelerometer and gyroscope, respectively. *b*_*a*_ and *b*_*w*_ represent the resolutions (in bits) of the accelerometer and gyroscope, corresponding to the number of quantization levels used to digitize the analog signal.

The BNO055 and MPU9250 IMUs were configured with the following parameters: the BNO055 accelerometer was set with a range of ±8g and a resolution of 14 bits, while the MPU9250 accelerometer operated at ±16g with a resolution of 16 bits. For both devices, the gyroscopes were configured with a range of ±2000°/*s* and a resolution of 16 bits.

#### 2.2.2 Definition of cow behaviors

The behaviors of interest for the dataset are walking, grazing, and resting, as these three are the most indicative of lameness-related changes in dairy cattle. Studies have shown that lame cows typically reduce their walking activity (8), spend less time grazing (6, 7), and increase their resting time as a compensatory strategy (9). Therefore, continuous monitoring of these behaviors provides valuable insight for early lameness detection.

To build a more robust and representative dataset, it is also necessary to label other less frequent behaviors such as standing, licking, shaking, head nodding, scratching, and rising. Including these actions helps prevent misclassification and allows the model to distinguish between major locomotor behaviors and isolated secondary activities. Table 1 provides the definition of each behavior analyzed in this study.

**Table 1:** Definitions of behaviors labeled in the dataset.

**Behavior**	**Definition**
Walking	Continuous movement at a moderate pace, starting when the animal takes its first step and ending when it stops with the last step.
Grazing	This behavior begins when the cow lowers its head to the ground and starts consuming grass and ends when it raises its head, stopping food intake.
Resting	The cow lies on the ground. This behavior starts when the cow lies down and ends when it gets up to resume activities.
Standing	A stationary position where the cow stands on all four legs without moving.
Drinking	Begins when the cow lowers its head to the water and starts drinking, and ends when it lifts its head away from the trough.
Shaking	A sudden and quick movement of the entire body.
Head nodding	A swaying or jerking motion of the head.
Licking	The cow uses its tongue to clean different parts of its body.
Rising	The animal transitions from lying down to standing. It begins when the cow pushes with its front or back legs and ends when it achieves a fully upright posture.
Lying down	The animal lowers its body to lie on the ground. It starts when the cow bends its legs and lowers its body, ending when it is fully recumbent.
Trotting	Rapid movement, starting when the cow significantly increases its speed compared to walking and ending when it stops or returns to a moderate pace.
Scratching	Action in which the animal uses a part of its body or objects from its environment to relieve itching.

The machine learning models in this work classify only four categories: *walking, grazing, resting*, and *miscellaneous behaviors*. The *miscellaneous behaviors* class groups all remaining behaviors that are not included in the three main categories, following a one-vs-rest strategy commonly used in behavior recognition tasks.

#### 2.2.3 Behavior labeling

The labeling of behaviors was based on the visual inspection of video recordings obtained from the PTZ cameras installed on the farm. The first step involved visually identifying the cow wearing the IoT collar. Since the cow's local identification number was not visible in the footage, photographs were taken during collar installation to document each cow's unique spot pattern. These spot patterns allowed the labeling team to match the cow observed in the video with the corresponding dataset collected by the collar.

Once the individual cow had been identified, the behaviors it exhibited were annotated by marking the start and end times of each event in an Excel file, along with other relevant information. As cow behavior is naturally variable in duration, the labeled events reflect non-uniform time lengths.

During the labeling process, it was observed that the timestamps of the collar data were not synchronized with the video timestamps; therefore, these discrepancies were identified and corrected through exhaustive event review. The synchronization between the video recordings and the signal data was performed manually by simultaneously analyzing both the video footage and the acceleration signals. This procedure was carried out only after accurately identifying the cow and confirming the specific collar it was wearing, ensuring that the observed behavior in the video corresponded precisely to the signal data recorded by that collar at that moment.

The labeling process was carried out by two research assistants and was supported by a veterinarian, who held periodic meetings with the team to validate each annotated behavior.

#### 2.2.4 Dataset structure

**Figure 5:**
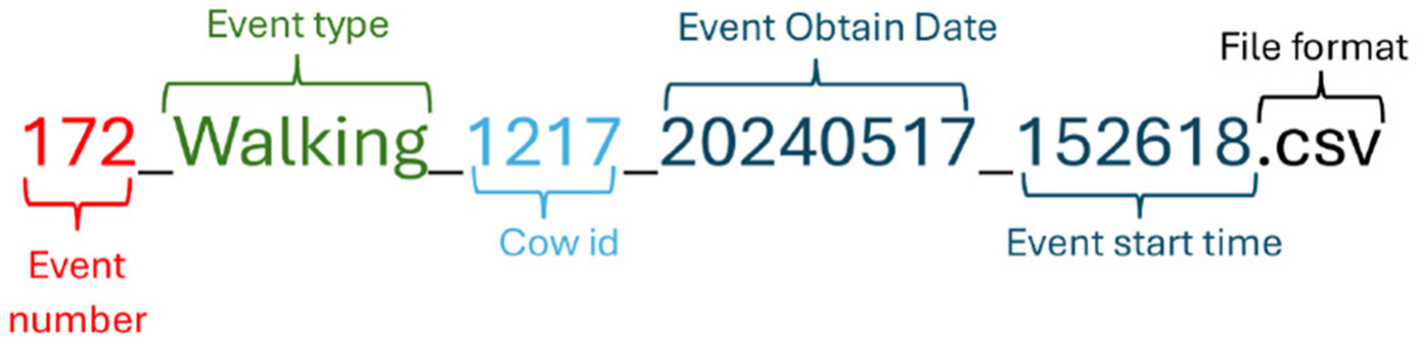
Structured format of CSV file names. The name consists of six elements: event number, event type, cow identifier, event acquisition date (YYYYMMDD), start time (HHMMSS), and file extension (.csv).

The recorded and labeled data were stored in individual CSV files, each corresponding to a single annotated behavior event. The naming convention of these files follows a standardized format that encodes key metadata, including the event number, behavior type, animal identifier, acquisition date, and start time, as illustrated in Figure 5. This facilitates systematic identification, traceability, and automated parsing of events.

**Figure 6:**
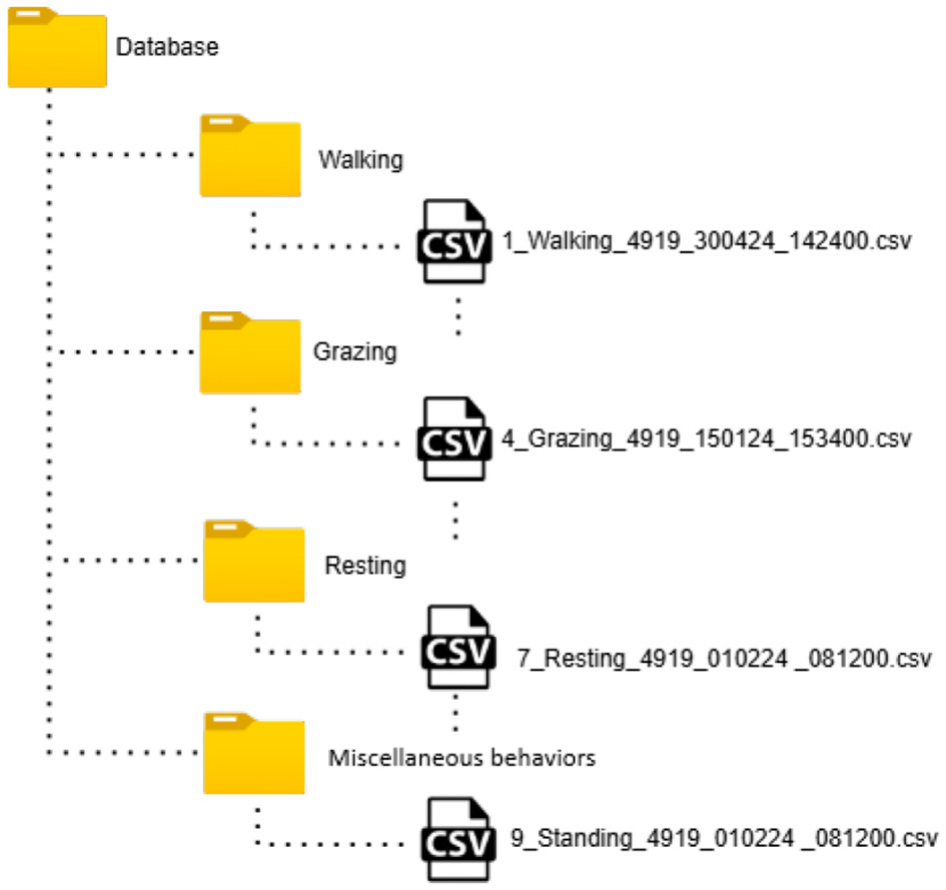
Hierarchical folder structure of the dataset. Each level represents a logical organization for storing and accessing the data.

To further enhance accessibility and organization, the dataset is arranged hierarchically into folders based on behavior categories, as shown in Figure 6. Each folder contains only the CSV files associated with a specific behavior, enabling streamlined filtering during model training.

Each CSV file contains time-series sensor data collected by the IMU collars. Table 2 provides a summary of the variables included, their units of measurement, and their corresponding column positions. The dataset integrates signals from two types of IMUs (BNO055 and MPU9250), offering redundancy. Variables include linear acceleration in both world and body reference frames, angular velocity, magnetometer data, and orientation quaternions.

**Table 2:** Summary of IMU variables, units of measurement, and corresponding column numbers.

**IMU**	**Variables**	**Unit of measurement**	**Column no**.
	Time (date and hour)	YYYY-MM-DD hh:mm:ss	1
BNO055	Linear acceleration [WF] (x,y,z)	*m*/*s*^2^	2:4
	Linear acceleration [BF] (x,y,z)	*m*/*s*^2^	5:7
	Gyroscope measurement (x,y,z)	°/*s*	8:10
	Magnetometer measurement (x,y,z)	-	11:13
	Orientation (q1,q2,q3,q4)	-	14:17
MPU9250	Linear acceleration [BF] (x,y,z)	*m*/*s*^2^	18:20
	Gyroscope measurement (x,y,z)	°/*s*	21:23
	Magnetometer measurement (x,y,z)	-	24:26

This structure was designed to support the practical needs of the project, which involved collecting multi-sensor data from several cows over an extended period in a real grazing environment. The file naming convention and folder organization facilitate systematic access, integration with video annotations, and automation of the preprocessing pipeline. It also ensures that each event is uniquely identifiable and reproducible, which is essential for future expansion of the dataset or replication by other research groups.

#### 2.2.5 Handling labels with outliers

Following the methodology described by Voss et al. (22), a statistical analysis of the data was conducted. Data points greater than three standard deviations from the mean were considered outliers.

This same methodology was used to identify and correct events that were mislabeled or erroneously recorded. To this end, the z-score statistical measure was used, which indicates how many standard deviations a specific value is above or below the mean. Labels with acceleration mean values for each axis with a z-score greater than 3 or less than −3 were considered outliers (beyond three standard deviations from the mean). Each of these outlier labels was reviewed and either removed or corrected in cases of misclassification of movements.

### 2.3 Machine learning algorithms

#### 2.3.1 Definition of the time window

**Figure 7:**
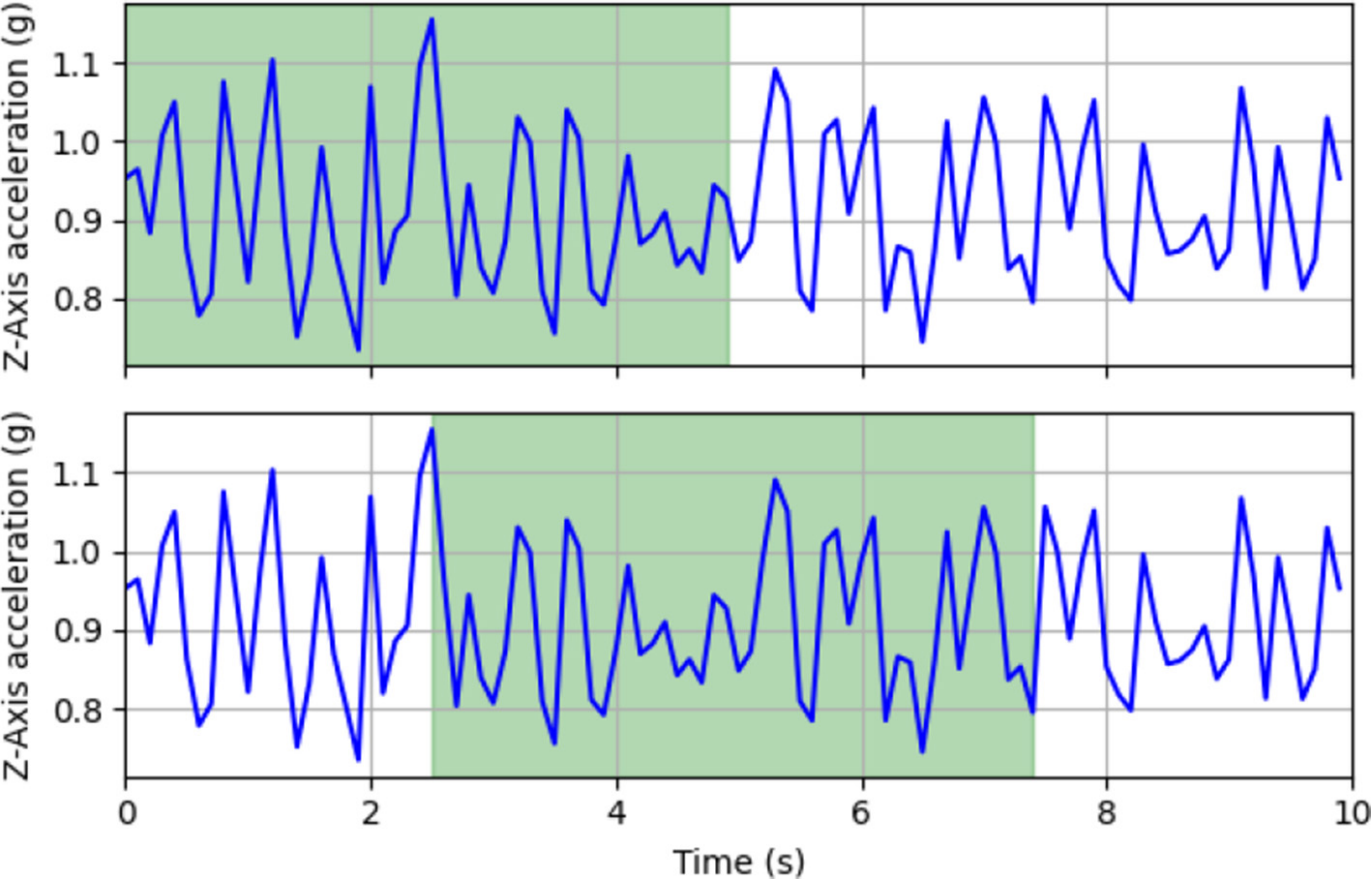
Example of 5-s window segmentation with 50% overlap (2.5 s) for a walking label.

The literature indicates that larger time windows, close to 30 seconds, yield better results in behavior classification (17, 23). However, choosing such a large time window would significantly reduce the dataset size. According to Wang et al. (24), a window of at least 5 s can adequately represent each behavior. Considering this, a time window of 5 s (50 samples) with a 50% overlap was selected for this study to segment the labels of each behavior. Figure 7 shows an example of how a walking label is segmented.

#### 2.3.2 Proposed features for classification

Feature extraction from acceleration measurements has been shown to yield good results. However, including gyroscope measurements could significantly improve behavior classification. This is evidenced by Liang et al. (17), where the most relevant features for classification were those derived from gyroscope measurements.

For this reason, in this study, the measurements of the linear acceleration in the *x*, *y*, and *z* axes in the body reference frame (IoT collar), the linear acceleration in the world reference frame, and the gyroscope measurements in the body reference frame (IoT collar) were used for feature extraction. Additionally, three derived signals were calculated using the following formulas:


(3) |wa|=awx2+awy2+awz2

(4) |ba|=abx2+aby2+abz2

(5) |bω|=ωbx2+ωby2+ωbz2

Where axw,ayw,azw represent the linear acceleration in the world reference frame along the *x*, *y*, and *z* axes. Similarly, axb,ayb,azb represent the linear acceleration in the body reference frame, and ωxb,ωyb,ωzb correspond to the gyroscope measurements in the body reference frame. ^*w*^|*a*| and ^*b*^|*a*| are the magnitude vectors of the acceleration in the world and body reference frames, respectively. ^*b*^|ω| is the magnitude of the gyroscope measurements.

Two datasets were defined: the first, called Dataset BF, is based solely on the body reference frame, while Dataset WF includes the linear acceleration data transformed into the world reference frame.

For each dataset (BF and WF), 14 features were computed for each of the 8 variables, resulting in a total of 112 features used for model training and validation. These features are listed in Table 3.

**Table 3:** Names of the features used for model training, along with the corresponding data sources.

**Feature name**	**Data**	
	**Dataset BF**	**Dataset WF**
Mean	axw,ayw,azw, axw,ayw,azw	axw,ayw,azw, axw,ayw,azw
Median		
Standard deviation		
Zero-crossing rate		
Peak to peak		
Sum		
Absolute sum		
Root mean square		
Average acceleration variation (AAV)		
Skewness		
Kurtosis		
Dominant frequency		
Dominant spectral density		
Average spectral density		
**Total**	**112**	**112**

Each feature was computed for all eight variables in both datasets: body frame and world frame. *a*: Linear acceleration measurements, ω: gyroscope measurements, subindex for *x*, *y*, and *z* axes; upperindex *b* for body frame and ω for world frame, and |  |: magnitude of the vector, i.e., $|a|=\sqrt{a_{x}^{2}+a_{y}^{2}+a_{z}^{2}}$.

#### 2.3.3 Accessing the dataset

To facilitate the application of the dataset in machine learning pipelines, a practical workflow was designed for reading, processing, and training models with the labeled behavioral data. Each CSV file contains time-series measurements for a single annotated event, and these files are organized by behavior and naming convention to support batch processing (as described previously).

The dataset can be used to generate feature vectors suitable for input into classification algorithms. A typical process involves reading each file, extracting statistical or spectral features from the accelerometer and gyroscope signals, assigning a class label, and constructing the corresponding input matrix.

Below is an example script written in Python that demonstrates how to load a single labeled file, extract basic features (mean and standard deviation), and train a simple classifier using scikit-learn:

**Algorithm 1:** Example of using the dataset for classification.

import pandas as pd
**import** numpy as np
**from** sklearn.model_selection **import** train_test_split
**from** sklearn.ensemble **import** RandomForestClassifier
**from** sklearn.preprocessing **import** StandardScaler
**from** sklearn.metrics **import** classification_report
# Load one CSV file (replace with a loop over all files)
df = pd.read_csv(~172_walking_1217_20240401_134500.csv~)
# Extract features (example: mean and std of x-acc and z-gyro)
features = [
df[~acc_x_body~].mean(), df[~acc_x_body~].std(),
df[~gyro_z~].mean(), df[~gyro_z~].std()
]
X = np.array([features])
Y = np.array([0]) # example label (e.g., 0 = walking)
# Train/test split
X = StandardScaler().fit_transform(X)
X_train, X_test, Y_train, Y_test = train_test_split(X, Y, test_size=0.2)
# Train classifier
clf = RandomForestClassifier()
clf.fit(X_train, Y_train)
pred = clf.predict(X_test)
**print**(classification_report(Y_test, pred))

This simplified example can be expanded to include all labeled files, additional features (such as frequency-domain metrics), and more complex models. A more complete implementation, including batch processing, feature extraction, and classifier evaluation, is available in the public repository at https://github.com/WASP-lab/db-cow-walking. The dataset's structure and consistent formatting allow for seamless integration with most machine learning workflows.

#### 2.3.4 Feature selection

Feature selection is a key step to improving the performance of machine learning models. By choosing a smaller and more relevant subset of features, greater accuracy can be achieved by avoiding irrelevant and redundant patterns. This also reduces training time and computational costs, as the model works with fewer variables. Eliminating unnecessary features enhances efficiency and accuracy, making this process an essential tool for optimizing machine learning.

Liang et al. (17) presents a machine learning approach based on IMU data for recognizing daily behavior patterns in dairy cows. This study used two feature selection techniques: Permutation Importance and Sequential Backward Selection (SBS).

Permutation Importance is a model-agnostic technique that evaluates the contribution of each feature by measuring the decline in model performance when the feature's values are randomly permuted. A significant drop in the F1-score indicates that the feature plays an important role in classification. Sequential Backward Selection is a greedy algorithm that starts with the complete set of features and removes the least relevant one in each iteration, based on its impact on model performance. These methods are commonly used to reduce dimensionality and improve generalization. In the work by (17), which focused on behavior recognition using IMU signals, both techniques were applied to identify relevant features from an initial set of 70. Their results showed that reducing to 58 features maintained a high F1-score of 0.87, and further reduction to 31 features still yielded 0.8506, demonstrating the effectiveness of these selection strategies.

In our study, after the training process, the trained model was evaluated using a test set (20% of the original dataset) to generate a baseline metric (in this case, the F1-score). Then, a feature from the test set was selected and permuted (its values were randomly rearranged), and predictions were made using the test set with the permuted feature. Feature importance was calculated as the difference between the baseline performance and the performance after permutation. This procedure was repeated for each feature in the test set, generating a list of relative importances. Features were then ranked in descending order of importance.

Finally, the Sequential Backward Selection technique was used. In this process, the algorithm was initially trained with all available features. In each iteration, the least important feature was removed, and the model's performance was evaluated using the F1-score for each resulting subset. At the end, the subset of features providing the best F1-score was selected, optimizing the model.

#### 2.3.5 Training the machine learning algorithms

To evaluate the utility of the dataset, four widely used supervised machine learning algorithms were implemented and trained: Support Vector Machine (SVM), Logistic Regression (LR), Decision Trees (DT), and Random Forest (RF). All models were developed in Python (version 3.11.9) using the scikit-learn library (version 1.5.1), which provides a consistent and efficient framework for model training, evaluation, and hyperparameter tuning.

Support Vector Machine (25) aims to find an optimal hyperplane that separates the data into distinct classes, maximizing the margin between the closest points of each class, known as support vectors. It works with both linearly separable and non-linear data using kernels. There are different ways to handle multiple classes using SVM, such as the One-vs-Rest and One-vs-One methods. This study uses a multi-class SVM with the One-vs-One approach, as it generally provides better performance. This method involves training a binary classifier for each pair of classes, and the results of each classifier are combined using a voting scheme (the class with the most votes is the final prediction).

Logistic Regression (26) uses a linear combination of independent variables and applies the sigmoid (logistic) function to model the probability that an observation belongs to one of two classes. The Decision Tree algorithm (27) works by repeatedly splitting the data into subsets based on the feature that best separates according to a specific criterion, such as information gain or Gini impurity. Tree nodes represent decisions based on features, and the leaves represent the final predictions. Random Forest (28) is a machine learning algorithm consisting of a collection of independently trained decision trees, with predictions made by majority voting or averaging the predictions of individual trees.

For model training and evaluation, the original dataset was randomly divided, with 80% assigned to the training set and 20% to the test set. Hyperparameter tuning for the models was conducted using the Grid Search method with k-fold cross-validation to ensure that overfitting is minimized or avoided altogether.

#### 2.3.6 Performance indices

The models' performance was evaluated using the metrics accuracy, precision, recall, F1-score, and macro-averaged F1-score. Accuracy is the percentage of correct predictions overall, representing how often the model was correct compared to all predictions. Precision is the proportion of correctly identified positive cases relative to all cases classified as positive. Recall is the proportion of positive cases correctly identified by the model relative to all actual positive cases. The F1-score is the harmonic mean of precision and recall, especially valuable for imbalanced datasets. The macro F1-score is the arithmetic mean of all F1 scores for each class, providing a single number to describe the overall performance of the models. These metrics are calculated as follows:


(6) $$\begin{array}{l} \text{Accuracy}=\frac{TP+TN}{TP+TN+FP+FN} \end{array}$$

(7) $$\begin{array}{l} \text{Precision}=\frac{TP}{TP+FP} \end{array}$$

(8) $$\begin{array}{l} \text{Recall}=\frac{TP}{TP+FN} \end{array}$$

(9) $$\begin{array}{l} \text{F1-score}=2\cdot \frac{\text{Precision}\cdot \text{Recall}}{\text{Precision}+\text{Recall}} \end{array}$$

(10) $$\begin{array}{l} \text{Macro F1-score}=\frac{\sum_{i=1}^{n}{\text{F1-score}}_{i}}{n} \end{array}$$

Where *TP*, *TN*, *FP*, and *FN* represent the number of true positives, true negatives, false positives, and false negatives, respectively.

## 3 Results

### 3.1 Unprocessed data

#### 3.1.1 Variables

Data collection from the IMUs was carried out between May 10 and October 8, 2024, involving 10 dairy cows. The recorded variables are detailed in Table 2. All data were stored on both Google Drive and a dedicated server, resulting in a total of 10 *Runs*, each compressed in ZIP format. A *Run* refers to the complete data collected during the time a collar was attached to an individual cow: from installation through removal and data extraction. Each *run* folder, representing a specific cow during a given monitoring campaign, contributes to a cumulative total of over 3 GB of raw data.

#### 3.1.2 Recordings

A total of 150 video recordings were collected using PTZ cameras positioned to capture the animals' behavior during collar operation. Each recording lasted approximately one hour, with a file size of 1.7 GB, resulting in a total data volume of around 255 GB. These videos were subsequently used for behavior annotation.

### 3.2 Dataset

As detailed in Table 4, the dataset consists of 441 labels, totaling 7 h, 34 min, and 2 s of recording. It can be observed that the predominant behavior is *walking*, with a total of 217 events, while *resting* is the behavior with the fewest observations, with only 10 events. However, the total time recorded for *resting* amounts to 3 h, 11 min, and 20 s, significantly surpassing the time recorded for *walking*, which is 53 min and 2 s. This implies that during time window segmentation, the *resting* behavior corresponds to the majority class. A detailed breakdown of the behaviors included in the *miscellaneous behaviors* class is provided in Table 5, showing that the predominant behavior within this class is *standing*.

**Table 4:** Number of events, average duration, and total duration (hh:mm:ss) of the main behaviors.

**Behavior**	**No. of labels**	**Average duration**	**Total duration**
Walking	217	00:00:14	00:53:02
Grazing	105	00:01:19	02:18:39
Resting	10	00:19:08	03:11:20
Miscellaneous behaviors	109	00:00:36	01:11:01
**Total**	**441**	**–**	**07:34:02**

**Table 5:** Number of events, average duration, and total duration (hh:mm:ss) of behaviors classified as *miscellaneous behaviors*.

**Behavior**	**No. of labels**	**Average duration**	**Total duration**
Standing	59	00:01:06	01:05:12
Drinking	3	00:00:15	00:00:47
Licking	9	00:00:10	00:01:30
Scratching	5	00:00:07	00:00:38
Head Nodding	8	00:00:06	00:00:48
Lying Down	4	00:00:05	00:00:20
Rising	10	00:00:05	00:00:50
Shaking	8	00:00:05	00:00:41
Trotting	3	00:00:05	00:00:15
Total	109	–	01:11:01

**Figure 8:**
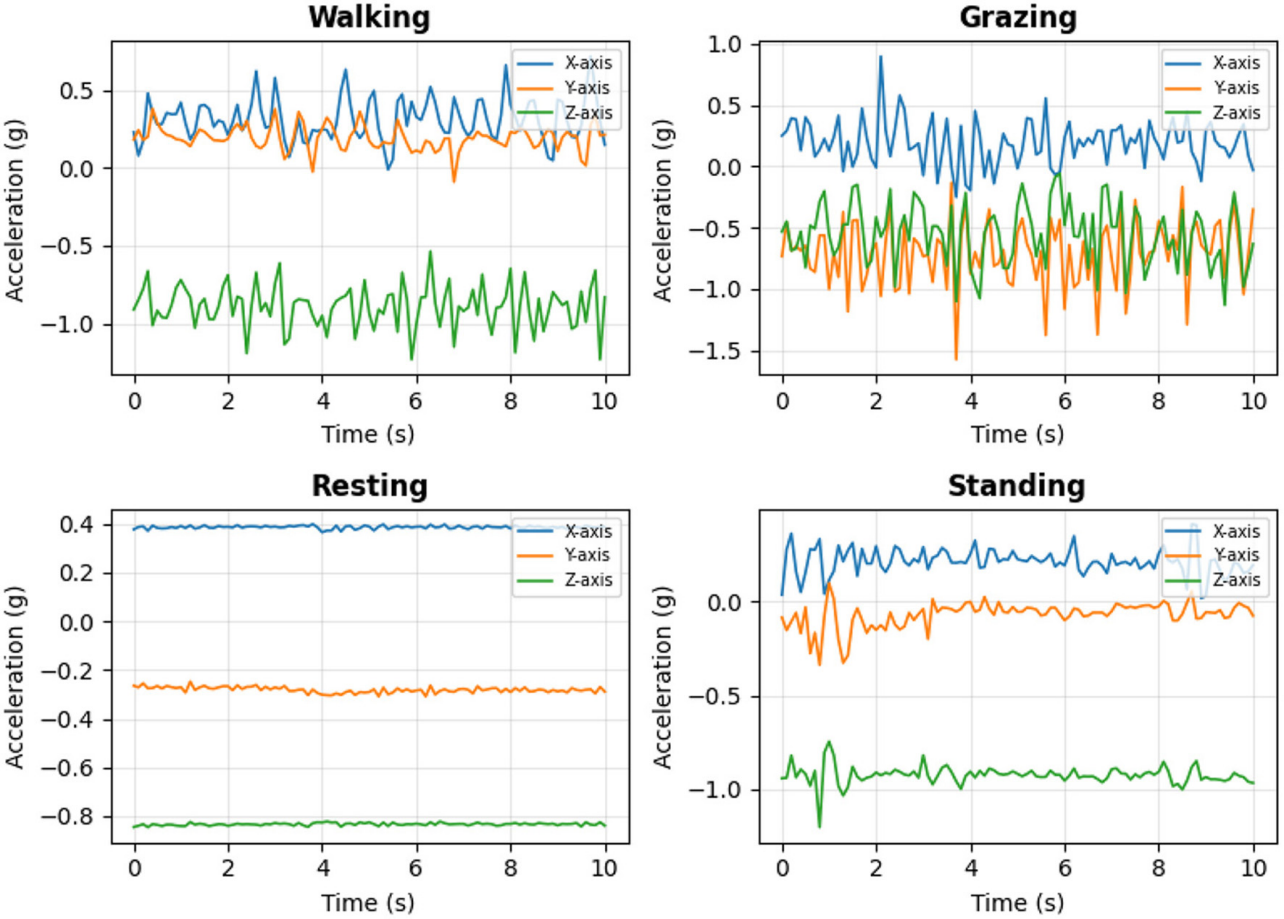
Graph of linear accelerations in the body reference frame (*x*, *y*, *z*) versus time for the four main behaviors: *walking, grazing, resting*, and *standing*.

Figure 8 shows the linear accelerations in the body reference frame for the *x*, *y*, and *z* axes for the four predominant behaviors. It can be observed that each behavior exhibits a distinct pattern. Activities such as walking and grazing show greater variability and amplitude in accelerations, while static activities such as resting and standing present more stable signals.

### 3.3 Evaluation of classification algorithms

The results presented in Table 6 were obtained after performing hyperparameter optimization using the Grid Search method with k-fold cross-validation, aiming to minimize or avoid overfitting and ensure optimal model performance. It can be observed that, overall, models achieve better performance for each behavior when trained with the dataset containing acceleration in the body reference frame. This indicates that acceleration in the world reference frame does not contribute to improving the classification of these behaviors. Furthermore, grazing achieves the best performance, with F1-scores of 0.99 in the best cases using support vector machine and logistic regression models, and an F1-score of 0.98 in the worst cases using decision tree and random forest models.

**Table 6:** Evaluation results for the different classification models using body and world reference frame accelerations.

**Model**	**Body accelerations**					**World accelerations**				
	**N**° **Features**	**Behavior**	**Precision**	**Recall**	**F1**	**N**° **Features**	**Behavior**	**Precision**	**Recall**	**F1**
**SVM**	39	Walking	0.89	0.94	0.92	40	Walking	0.82	0.85	0.84
		Grazing	0.99	0.99	0.99		Grazing	0.98	0.98	0.98
		Resting	0.96	0.98	0.97		Resting	0.92	0.97	0.95
		Miscellaneous	0.96	0.86	0.91		Miscellaneous	0.91	0.75	0.82
		behaviors					behaviors			
**LR**	39	Walking	0.88	0.85	0.86	39	Walking	0.84	0.79	0.81
		Grazing	0.98	1.00	0.99		Grazing	0.97	0.98	0.98
		Resting	0.96	0.98	0.97		Resting	0.92	0.97	0.95
		Miscellaneous	0.91	0.85	0.88		Miscellaneous	0.88	0.76	0.82
		behaviors					behaviors			
**DT**	22	Walking	0.89	0.83	0.86	20	Walking	0.75	0.68	0.71
		Grazing	0.96	0.99	0.98		Grazing	0.92	0.95	0.94
		Resting	0.93	0.97	0.95		Resting	0.92	0.96	0.94
		Miscellaneous	0.89	0.77	0.82		Miscellaneous	0.88	0.74	0.81
		behaviors					behaviors			
**RF**	35	Walking	0.87	0.92	0.89	38	Walking	0.88	0.59	0.71
		Grazing	0.97	0.99	0.98		Grazing	0.88	0.98	0.93
		Resting	0.96	0.98	0.97		Resting	0.91	0.98	0.95
		Miscellaneous	0.96	0.82	0.89		Miscellaneous	0.93	0.69	0.79
		behaviors					behaviors			

**SVM**, Support Vector Machine; **LR**, Logistic Regression; **DT**, Decision Tree; **RF**, Random Forest.

Table 7 summarizes the evaluation results for the models, showing the accuracy and macro F1-score metrics for each model and dataset. For models trained with the dataset containing acceleration in the body reference frame, the highest macro F1-score was 0.9625 with the support vector machine model. The random forest and logistic regression models also performed well, achieving macro F1-scores of 0.9539 and 0.9509, respectively. The model trained with the decision tree method presented the lowest performance, with a macro F1-score of 0.9290. Additionally, grazing and resting behaviors achieved the best performance across all evaluation metrics, while walking and others showed the lowest performance.

**Table 7:** Summary of model evaluation results for each dataset.

**Model**	**Body accelerations**		**World accelerations**	
	**Accuracy**	**Macro F1 score**	**Accuracy**	**Macro F1 SCORE**
SVM	0.9629	0.9625	0.9292	0.8965
LR	0.9517	0.9509	0.9248	0.8879
DT	0.9307	0.9291	0.8994	0.8489
RF	0.9546	0.9539	0.9004	0.8430

**SVM**, Support Vector Machine; **LR**, Logistic Regression; **DT**, Decision Tree; **RF**, Random Forest.

For machine learning models trained with the dataset including acceleration in the world reference frame, the best macro F1-score was 0.8965 with the SVM model, while the worst performance was achieved by the random forest model, with a macro F1-score of 0.8430.

These results demonstrate the usefulness of the proposed dataset for classifying key dairy cow behaviors under open grazing conditions, paving the way for future detection systems targeting early signs of lameness.

## 4 Discussion

Several studies have explored the classification of cattle behaviors or the detection of lameness using IMU-based data. For instance, Ismael et al. (10) proposed CowScreeningDB, a multisensor dataset collected from 43 confined cows, using an SVM model that achieved up to 77% accuracy in identifying lame individuals. Similarly, Haladjian et al. (11) proposed a sensor-based system for the same purpose, collecting data from 10 cows and achieving a higher average accuracy of 91.1%. The results obtained by Ismael et al. (10) and Haladjian et al. (11) are not directly comparable to the present work, as this research focuses on developing a dataset of behaviors associated with lameness rather than directly identifying a lame cow.

In addition, Ito et al. (14) presented a dataset including triaxial accelerometer data from six Japanese black cows with 13 labeled behaviors. The dataset was collected under open grazing and pen conditions using neck-mounted sensors and manual video labeling. Russel et al. (15) used this dataset to train deep learning models, achieving up to 0.99 F1-score. Cabezas et al. (29) also obtained classification accuracies above 0.93 using Random Forest models and neck-mounted accelerometers. While these results are strong, those datasets either lacked gyroscope measurements or were not collected continuously in extensive pasture conditions.

Compared to the present study, the work by Versluijs et al. (18) stands out for its use of orientation-dependent features, i.e., accelerations measured in the body reference frame, to classify behaviors of free-ranging beef cattle in forested pastures in Norway. Their best-performing model used a 20-second smoothing window and achieved excellent results: an accuracy of 0.997, precision of 0.961, and recall of 0.985. However, there are key methodological differences. Versluijs et al. recorded behavioral data using handheld Canon cameras, requiring close human presence and potentially influencing animal behavior. In contrast. Our system employed PTZ IP cameras mounted on elevated poles with GPS-guided framing, enabling continuous monitoring without disturbing the animals, thus avoiding the presence of humans in the video recording process and preserving natural behavior patterns.

Another major distinction lies in the sensing hardware. Versluijs et al. used commercial virtual fencing collars named “Nofence” with built-in IMUs, whereas our custom-designed collar is intended to be low-cost and modular. It integrates two independent IMUs (BNO055 and MPU9250), allowing redundancy and cross-validation of sensor data. Additionally, our system collects both linear acceleration and angular velocity.

From an ethological standpoint, our study focused on dairy cows in an open grazing system, while Versluijs et al. analyzed the behavior of beef cattle. These differences in management system and breed type may also influence the behavioral repertoire and sensor signal profiles. Despite these contrasts, both studies demonstrated that body-frame accelerations yield high classification performance. Notably, while Versluijs et al. applied a simple manual correction (axis inversion when collars were mounted backward), we implemented continuous orientation correction using quaternion-based sensor fusion with magnetometer and accelerometer inputs, transforming data to a world reference frame. However, this correction did not improve performance in our case, likely due to magnetometer anomalies.

During the study, it was observed that signals captured by the magnetometer exhibited certain anomalies in amplitude, affecting orientation estimates and, consequently, the calculation of accelerations in the world reference frame. This could explain why these accelerations performed worse than those in the body reference frame.

In the best case, using an SVM model trained with body-frame accelerations and gyroscopic data, an F1-score of 0.9625 was achieved. This indicates that even with basic classification algorithms, robust results can be obtained when high-quality labeled data is available. Although this value is slightly lower than the 0.99 F1-score achieved by Russel et al. (15), their results were obtained using deep learning techniques on a smaller and less diverse dataset. Notably, the grazing behavior achieved the highest F1-score in our study, reaching 0.99 with the SVM model. This behavior is particularly relevant, as it is directly linked to milk production; thus, accurate monitoring can contribute to improved management decisions and increased economic returns (30).

Although this study does not directly address lameness detection and no lame cows were included in the dataset, the resulting data and findings are relevant from a lameness detection perspective. It is well established that lameness in dairy cows is associated with behavioral changes such as reduced walking activity, decreased grazing time, and increased resting periods. In this context, the classification algorithms developed in this study successfully identified different types of behavior, suggesting that models trained on this dataset could serve as a foundation for monitoring systems aimed at detecting early signs of lameness.

## 5 Conclusions

This study developed a dataset aimed at identifying behaviors associated with lameness in dairy cows. The dataset contains linear acceleration and angular velocity signals, totaling 441 labels and 7 hours, 34 minutes, and 2 seconds of recordings corresponding to 11 different behaviors. The data were collected from 10 cows grazing in an open pasture system over a period of approximately five months. The entire dataset generation process is fully documented, characterized, and verified, ensuring data reliability and result reproducibility. To test the dataset's adequacy, machine learning algorithms were trained. In general, the models achieved better performance when trained with the dataset composed of body reference frame accelerations along with angular velocity. In the best case, using an SVM model, an F1-score of 0.9625 was achieved. Although this performance is lower than the 0.99 F1-score obtained by Russel et al. (15) using deep learning models, only basic classification algorithms were used in this work. This also confirms that accelerations in the world reference frame do not contribute to improving the classification of these behaviors.

A limitation of this study is the low number of cows used to build the dataset. With only 10 individuals, the results may not fully capture the behavioral variability in larger or more diverse dairy populations. This limitation may affect the generalization of the trained models and highlights the need for future studies with larger and more representative samples. This limitation highlights the potential benefits of expanding the dataset in future studies. The open pasture dairy cow behavior dataset is made publicly available for use in training classification models. It can be further enhanced by increasing the number of labels for each behavior and the number of cows studied. This would allow the creation of more robust and representative models by capturing a greater diversity of data, reducing biases, and improving generalization across different contexts. Moreover, a larger dataset would facilitate using more advanced algorithms, including deep learning techniques. Increasing the duration of behavior recordings is also important for evaluating the effect of different time window sizes and identifying optimal configurations for classification tasks.

In conclusion, this work contributes a well-documented and publicly available dataset of dairy cow behaviors under open grazing conditions. The data and classification results presented here support the development of reliable, scalable monitoring systems aimed at improving animal welfare and early detection of lameness in real-world pasture environments.

## Data Availability

The datasets presented in this study can be found in online repositories. The names of the repository/repositories and accession number(s) can be found below: https://github.com/WASP-lab/db-cow-walking.
